# Retraction: The WNK-regulated SPAK/OSR1 kinases directly phosphorylate and inhibit the K+-Cl- co-transporters

**DOI:** 10.1042/BCJ20131478_RET

**Published:** 2025-12-17

The authors of the article “The WNK-regulated SPAK/OSR1 kinases directly phosphorylate and inhibit the K+-Cl- co-transporters” (DOI: 10.1042/BJ20131478) wish to retract the article from the *Biochemical Journal* after being alerted by a reader to multiple errors across Figures 3–7 of the published work.

These errors included misalignment of digitised Western blot data, incorrect figure labelling and instances of blot duplication due to miscommunication between laboratory members involved in preparing the figures following peer review. Unfortunately, these errors were not detected during the final pre-publication review.

The authors have located and verified the original raw data and laboratory notebooks for all experiments. These data reaffirm the study’s central conclusion, that WNK-regulated SPAK/OSR1 kinases directly phosphorylate and inhibit K+-Cl- co-transporters. These findings have been independently replicated and supported by numerous peer-reviewed publications over the past decade, and the phospho-specific antibodies generated in this study have also been widely used to investigate KCC regulation (e.g. DOI: 10.1074/jbc.m117.817841; DOI: 10.1074/jbc.m705095200; DOI: 10.1073/pnas.1810134115; and DOI: 10.1038 /s41467-019-13851-6). The authors therefore stand by the conclusions of the paper.

However, given the number of errors identified, and in the interest of maintaining scientific transparency and integrity of the published record, the authors collectively agree that retraction of this article is the most appropriate course of action.

The Publisher reviewed original data provided by the authors and agrees with retraction of the paper.

The authors sincerely apologise to the editors, reviewers and readers of the *Biochemical Journal* for the inconvenience caused by these unintentional errors. The authors intend to repeat the key experiments to verify the findings, and the confirmed results will be submitted for consideration of publication in due course.

